# Monitoring tumor cell death in murine tumor models using deuterium magnetic resonance spectroscopy and spectroscopic imaging

**DOI:** 10.1073/pnas.2014631118

**Published:** 2021-03-16

**Authors:** Friederike Hesse, Vencel Somai, Felix Kreis, Flaviu Bulat, Alan J. Wright, Kevin M. Brindle

**Affiliations:** ^a^Cancer Research UK Cambridge Institute, University of Cambridge, Cambridge CB2 0RE, United Kingdom;; ^b^Department of Radiology, School of Clinical Medicine, University of Cambridge, Cambridge CB2 0SL, United Kingdom;; ^c^Department of Information Technology and Electrical Engineering, ETH Zürich, 8092 Zürich, Switzerland;; ^d^Department of Chemistry, University of Cambridge, Cambridge CB2 1EW, United Kingdom;; ^e^Department of Biochemistry, University of Cambridge, Cambridge CB2 1GA, United Kingdom

**Keywords:** cell death, tumor, MRI, deuterium

## Abstract

^2^H magnetic resonance spectroscopic imaging has been shown recently to be a viable technique for metabolic imaging in the clinic. We show here that ^2^H MR spectroscopy and spectroscopic imaging measurements of [2,3-^2^H_2_]malate production from [2,3-^2^H_2_]fumarate can be used to detect tumor cell death in vivo via the production of labeled malate. Production of [2,3-^2^H_2_]malate, following injection of [2,3-^2^H_2_]fumarate (1 g/kg) into tumor-bearing mice, was measured in a murine lymphoma (EL4) treated with etoposide, and in human breast (MDA-MB-231) and colorectal (Colo205) xenografts treated with a TRAILR2 agonist, using surface-coil localized ^2^H MR spectroscopy at 7 T. Malate production was also imaged in EL4 tumors using a fast ^2^H chemical shift imaging sequence. The malate/fumarate ratio increased from 0.016 ± 0.02 to 0.16 ± 0.14 in EL4 tumors 48 h after drug treatment (*P* = 0.0024, *n* = 3), and from 0.019 ± 0.03 to 0.25 ± 0.23 in MDA-MB-231 tumors (*P* = 0.0001, *n* = 5) and from 0.016 ± 0.04 to 0.28 ± 0.26 in Colo205 tumors (*P* = 0.0002, *n* = 5) 24 h after drug treatment. These increases were correlated with increased levels of cell death measured in excised tumor sections obtained immediately after imaging. ^2^H MR measurements of [2,3-^2^H_2_]malate production from [2,3-^2^H_2_]fumarate provide a potentially less expensive and more sensitive method for detecting cell death in vivo than ^13^C MR measurements of hyperpolarized [1,4-^13^C_2_]fumarate metabolism, which have been used previously for this purpose.

## Significance

There is an unmet clinical need for sensitive methods for detecting cell death in vivo, for example, in disease and following tumor treatment. We show here that deuterium magnetic resonance measurements at 7 T of labeled malate production from injected ^2^H-labeled fumarate provide a sensitive method for detecting tumor cell death in vivo following treatment. Malate production was relatively slow in viable cells but was markedly increased in necrotic tissue.

Currently, the response of solid tumors to treatment is assessed mainly on the basis of changes in tumor size [Response Evaluation Criteria in Solid Tumors (RECIST) (1)]. However, changes in size may take weeks to appear after the initiation of treatment and, in some cases, may not appear at all, for example, in the case of treatments that inhibit tumor growth but do not result in tumor regression (2, 3). Changes in metabolism can give an earlier indication of treatment response, for example, assessment of glycolytic activity using positron emission tomography (PET) measurements of 2-[^18^F]-fluoro-2-deoxy D-glucose uptake (FDG-PET). PET Response Criteria in Solid Tumors (PERCIST) was introduced as a potentially more sensitive method of assessing treatment response when compared to assessment based on changes in tumor size alone (4), particularly with therapies that stabilize disease. Imaging with hyperpolarized [1-^13^C]pyruvate, like FDG-PET, can also be used to detect drug target engagement as well as subsequent tumor cell death (5, 6). We have shown recently that imaging hyperpolarized [1-^13^C]pyruvate metabolism can be more sensitive than FDG-PET in detecting reductions in glycolytic flux associated with tumor cell death posttreatment (7).

While metabolic imaging may indicate drug target engagement, and in some cases tumor cell death, there is a need for imaging methods that detect tumor cell death more directly posttreatment and that can give an indication of longer-term treatment outcomes (8). Fumarate is hydrated in the reaction catalyzed by the intracellular enzyme fumarase to produce malate. Previous ^13^C magnetic resonance spectroscopic imaging (MRSI) studies with hyperpolarized [1,4-^13^C_2_]fumarate in tumor models and in models of myocardial infarction and acute kidney necrosis (9–16) have demonstrated that the production of labeled malate can be used to image cell death in vivo. The increased production of malate was attributed to loss of the plasma membrane permeability barrier in necrotic cells and increased access of hyperpolarized [1,4-^13^C_2_]fumarate to fumarase. However, imaging with hyperpolarized ^13^C-labeled substrates is limited both by the transient nature of the hyperpolarization, which restricts its application to relatively fast metabolic processes, and the requirement for relatively large amounts of ^13^C-labeled compounds and access to clinical hyperpolarizers, which are expensive. The recent demonstration by De Feyter et al. that ^2^H MRSI can be used to image the metabolism of ^2^H-labeled substrates in vivo, including in human subjects (17), has provided a potentially lower-cost alternative for clinical metabolic imaging. The relatively low sensitivity of ^2^H detection is compensated by its very short T_1_, which means that signal can be acquired rapidly without saturation. The main limitation is the narrow frequency range, which requires the use of relatively high magnetic field strengths. Nevertheless, by collecting a series of rapidly acquired images, the technique is capable of generating quantitative images of metabolic flux (18). We show here that ^2^H magnetic resonance spectroscopy (MRS) and MRSI measurements of [2,3-^2^H_2_]fumarate conversion to ^2^H-labeled malate can be used to detect tumor cell death in vivo and that this is potentially a more sensitive method for detecting cell death than ^13^C MRSI with either hyperpolarized [1-^13^C]pyruvate or [1,4-^13^C_2_]fumarate.

## Materials and Methods

More detailed information is given in the *SI Appendix*.

### Cell Culture.

Murine lymphoma EL4 and human colorectal adenocarcinoma Colo205 cells were cultured in RPMI medium (Life Technologies) supplemented with 2 mM l-glutamine and 10% fetal bovine serum (FBS) (Gibco/Thermo Fisher Scientific) and human triple negative breast cancer MDA-MB-231 cells in Dulbecco’s Modified Eagle Medium (DMEM) (Gibco) containing 10% FBS.

### Tumor Implantation.

EL4, MDA-MB-231 or Colo205 cells were injected subcutaneously at 5 × 10^6^, 7 × 10^6^, and 10 × 10^6^ cells, respectively. EL4 tumors were allowed to develop for 10 d before imaging, whereas Colo205 and MDA-MB-231 tumors were imaged after 14 d and 35, d respectively. Animals bearing EL4 tumors were treated with etoposide (67 mg/kg of body weight, intraperitoneally) or solvent vehicle (saline) 48 h before imaging. Animals bearing MDA-MB-231 or Colo205 tumors were treated with MEDI3039 (19) (0.8 mg/kg of body weight, intravenously) or solvent vehicle (saline) 24 h before imaging. All animal experiments were carried out in compliance with project and personal licenses issued by the Home Office, UK, and approved by the Cancer Research UK, Cambridge Institute Animal Welfare and Ethical Review Body.

### Synthesis of Disodium [2,3-^2^H_2_]fumarate and Measurement of T_1_.

Three grams of [2,3-^2^H_2_]fumaric acid (Sigma-Aldrich) were dissolved in 8 M NaOH and then freeze-dried. The ^2^H T_1_s of fumarate, semi-heavy water (HDO), DMSO-d6 (3 mM), and formate-d (5 mM), which were used as chemical shift and intensity standards, were measured using an inversion recovery sequence at 147 ms, 199 ms, 719 ms, and 1.637 s, respectively.

### ^2^H MR Spectroscopy Measurements on Media Samples.

EL4 cells in suspension were treated with 15 μM etoposide and Colo205 and MDA-MB-231 cells on plates with 10 pM MEDI3039 for 24 h. The cells were then washed in PBS, resuspended at 1 × 10^6^ cells/mL in 10 mL culture medium, and 5 mM [2,3-^2^H_2_]fumarate was added. One-mililiter samples were taken at the specified time points, and ^2^H NMR spectra were acquired The amplitudes of the water, fumarate, and malate resonances were normalized to the DMSO-d6 peak, after correction for the slight saturation of the DMSO-d6 resonance, in order to calculate concentrations (17, 18). Absolute concentrations were obtained by correcting for the numbers of deuterons per molecule.

### MR Spectroscopy and Spectroscopic Imaging in Vivo.

Experiments were performed at 7 T (Agilent, Palo Alto, CA) using a 72 mm diameter birdcage volume coil for ^1^H transmit and receive (Rapid Biomedical GMBH, Rimpar, Germany) and a home-built 10 mm diameter single-loop surface coil, located over the tumor, for ^2^H transmit and receive. The tumors were localized in axial ^1^H images acquired with a fast spin echo (FSE) pulse sequence. Serial ^2^H spectra were acquired using a pulse-acquire sequence with a 2 ms BIR4 (20) adiabatic excitation pulse, with nominal flip angle of 67°, a repetition time (TR) of 140 ms, and a spoiler gradient on the *z* axis to dephase any residual transverse magnetization. Thirteen 5 min spectra were acquired over 65 min. Localization of signal to the tumors was achieved by the excitation profile of the surface coil and confirmed by chemical shift images, which showed that signal was largely confined to the tumors. After peak fitting, the intensity of the HDO peak at the first time point was assumed to correspond to the natural abundance of ^2^H in HDO, which has been estimated to be 10.12 mM in tissue (17, 18). The HDO signal provides an internal reference, which was used to estimate the concentration of deuterated fumarate. The signals were corrected for saturation (15%) by assuming that the T_1_s in vivo were similar to those measured in vitro at 14.1 T and correcting for the number of ^2^H nuclei in [2,3-^2^H_2_]fumarate. The concentration of malate was estimated from the intensity of the upfield resonance, assuming that it has a similar T_1_ to the fumarate resonance. The downfield resonance and the HDO peak overlap in vivo, which results in a slight overestimation of the labeled water concentration at later time points. Three-dimensional chemical shift images (CSI) were acquired as described in ref. 18. Disodium [2,3-^2^H_2_]fumarate was dissolved in water at a concentration of 312.5 mM and infused via a tail vein catheter. The infusion started 5 min after the start of spectral or image acquisition and resulted in 1 g/kg body weight of [2,3-^2^H_2_] disodium fumarate infused over a period of 20 min.

### MR Spectroscopy of Blood Extracts.

EL4 tumor-bearing mice (*n* = 6) were injected with 1 g/kg [2,3-^2^H_2_]fumarate under isoflurane anesthesia. Blood was taken via cardiac puncture at 20 (*n* = 3) and 70 (*n* = 3) min after fumarate injection. Blood from two more mice was taken without prior injection of fumarate. The blood was vortexed in ice-cold 2M perchloric acid (PCA) for 30 s, centrifuged at 13,000 g at 4 °C for 15 min and then neutralized with ice-cold 2M potassium hydroxide (KOH). The neutralized extract was then centrifuged for 10 min at 13,000 *g* and 200 µL of the supernatant added to 300 µL H_2_O and a formate-d standard added to a final concentration of 4 mM. ^2^H NMR spectra were acquired using the same acquisition parameters as used for the media samples but with a TR of 3 s. Concentrations were calculated by comparison of the signal intensities with that of the formate-d standard, after correction for slight signal saturation in the latter. A standard for proton NMR measurements (3-(trimethylsilyl)-2,2,3,3-tetradeuteropropionic acid) was then added to give a final concentration of 1 mM, together with 50 µL ^2^H_2_O, and ^1^H spectra were acquired with water presaturation and a flip angle of 90° into 16,384 data points, with a spectral width of 7,788 Hz and a repetition time of 8 s.

### MR Spectroscopy of Tissue Extracts.

EL4 tumor-bearing mice were injected with 1 g/kg [2,3-^2^H_2_]fumarate (*n* = 3) or PBS (*n* = 3) under isoflurane anesthesia, and after 20 min the animals were killed by cervical dislocation, and tumor, kidney, liver, and muscle tissue were freeze-clamped in liquid nitrogen-cooled tongs. For measurements on heart muscle, nontumor bearing C57BL/6J mice (*n* = 8) were injected with 1 g/kg [2,3-^2^H_2_]fumarate or PBS (*n* = 3) under isoflurane anesthesia, and after 20 (*n* = 3) and 60 min (*n* = 2) the animals were killed by cervical dislocation. The chest cavity was opened to expose the heart; the descending aorta, inferior vena cava, and pulmonary trunk were cut, and the heart was immediately flushed anterograde with ice-cold PBS by inserting a cannula into the apex of the right ventricle to inhibit contraction and to flush blood from the chambers. Remaining blood was aspirated from the ventricles, and the heart was submerged in a dish filled with ice-cold oxygenated Krebs–Henseleit buffer before the tissue was freeze-clamped in liquid nitrogen-cooled tongs. The frozen samples were homogenized in ice-cold 2M PCA using a Precellys Cryolys Evolution tissue homogenizer (Bertin Instruments) and neutralized with 2M KOH. After centrifugation for 15 min at 13,000 *g*, 200 µL of the supernatant was mixed with 300 µL of H_2_O, and a formate-d standard was added to a final concentration of 4 mM. ^2^H NMR spectra were acquired using the same acquisition parameters as used for the blood samples. Following the ^2^H NMR measurements, a standard for proton NMR measurements, 3-(trimethylsilyl)-2,2,3,3-tetradeuteropropionic acid was added at a final concentration of 1 mM, together with 50 µL of ^2^H_2_O, and ^1^H spectra were acquired as for the blood samples.

### ^2^H MR Spectroscopy of Erythrocyte Suspensions.

Erythrocytes were diluted to a hematocrit of ∼40% in Krebs–Henseleit buffer (NaCl 118.5; NaHCO_3_ 25.0; KCl 4.7; KH_2_PO_4_ 1.2; MgSO_4_ 1.2; glucose 11; CaCl_2_ 2.4; mmol/L, pH 7.4) gassed with 95% O_2_/5% CO_2_. ^2^H NMR spectra were acquired at 310 K following the addition of 8 mM [2,3-^2^H_2_]fumarate and 5 mM formate-d6. Spectra were acquired using a 90° pulse, a repetition time of 2 s, and were the sum of 150 transients.

### Dynamic Contrast-Enhanced MRI.

EL4, MDA-MB-231, and Colo205 tumor-bearing animals (*n* = 3 per group) underwent dynamic contrast-enhanced (DCE)-MRI before and either 48 (EL4) or 24 (MDA-MB-231 and Colo205) h after treatment with etoposide (EL4) or MEDI3039 (MDA-MB-231 and Colo205). Images were acquired at 9.4 T using a 40 mm diameter ^1^H volume coil. Baseline T_1_ measurements were made using an inversion recovery-FSE sequence. A series of 500 spoiled gradient echo images (2 averages, 5 s per set of 3 images) were acquired. Dotarem, at 200 μmol/kg (Gadoteric acid, Guerbet), was injected via a tail vein after the tenth image. Signals from the image series were converted, on a pixel-by-pixel basis, to a contrast-agent concentration by assuming an R_1_ relaxivity of the contrast agent of 2.7 s^-1^ ⋅ mM^−1^ (21).

### Western Blotting.

Freeze-clamped tumor samples were homogenized in 10 μL/mg radio-immunoprecipitation assay (RIPA) buffer (ThermoFisher Scientific) containing complete, mini ethylenediaminetetraacetic acid (EDTA)-free protease inhibitor (Sigma Aldrich). Membranes were probed with a rabbit polyclonal immunoglobulin G (IgG) fumarase antibody (ThermoFisher) and a mouse monoclonal glyceraldehyde 3-phosphate dehydrogenase (GAPDH) antibody (Abcam) and detected using IRDye 800CW Goat anti-Rabbit IgG (LI-COR) and IRDye 680LT Goat anti-Mouse IgG antibodies (LI-COR).

### Measurements of Fumarase Activity and ATP Concentration in Tissue Extracts.

Fumarase activity and adenosine triphosphate (ATP) concentrations were determined using colorimetric (ab196992, Abcam) and fluorometric kits (ab83355, Abcam), respectively. At 48 h after treatment with etoposide (EL4), and 24 h after treatment with MEDI3039 (MDA-MB-231 and Colo205) or drug vehicle, tumors were freeze-clamped and either homogenized in ice-cold 4 M PCA (ATP assay) or in assay buffer (fumarase assay) using a Precellys 24 homogenizer (Stretton Scientific). The ATP extracts were neutralized with 8 M KOH. Fumarase activity was assayed spectrophotometrically by measuring the conversion of malate to fumarate from the increase in absorbance at 450 nm, according to the manufacturer’s instructions. ATP concentration was determined fluorometrically (Ex/Em = 535/587 nm), again according to the manufacturer’s instructions. Both measurements were made using a PHERAstar FS microplate reader (BMG Labtech).

### Histology and Immunohistochemistry.

Sections of formalin-fixed paraffin-embedded tumors (10 μm) were stained with hematoxylin and eosin and with a rabbit monoclonal anti-CC3 antibody (Cell Signaling Technology) and a donkey anti-rabbit secondary biotinylated antibody (Jackson ImmunoResearch Laboratories). Sections were also stained using TdT-mediated dUTP Nick- End Labeling (TUNEL) (PromegaBenelux BV). Slides were scanned at 20× magnification with a resolution of 0.5 μm per pixel on an Aperio AT2 (Leica Biosystems).

### Statistical Analysis.

Statistical and graphical analyses was performed using Prism v8 (GraphPad). Data are shown as mean ± SD, unless stated otherwise. Analysis of variance was used for multiple comparisons of groups to determine significance. A paired or unpaired Student’s *t* test was used for single-parameter comparisons. *P* values are summarized in the figures.

## Results

### Deuterated Fumarate Metabolism Detects Tumor Cell Death In Vitro.

**Fig. 1.**
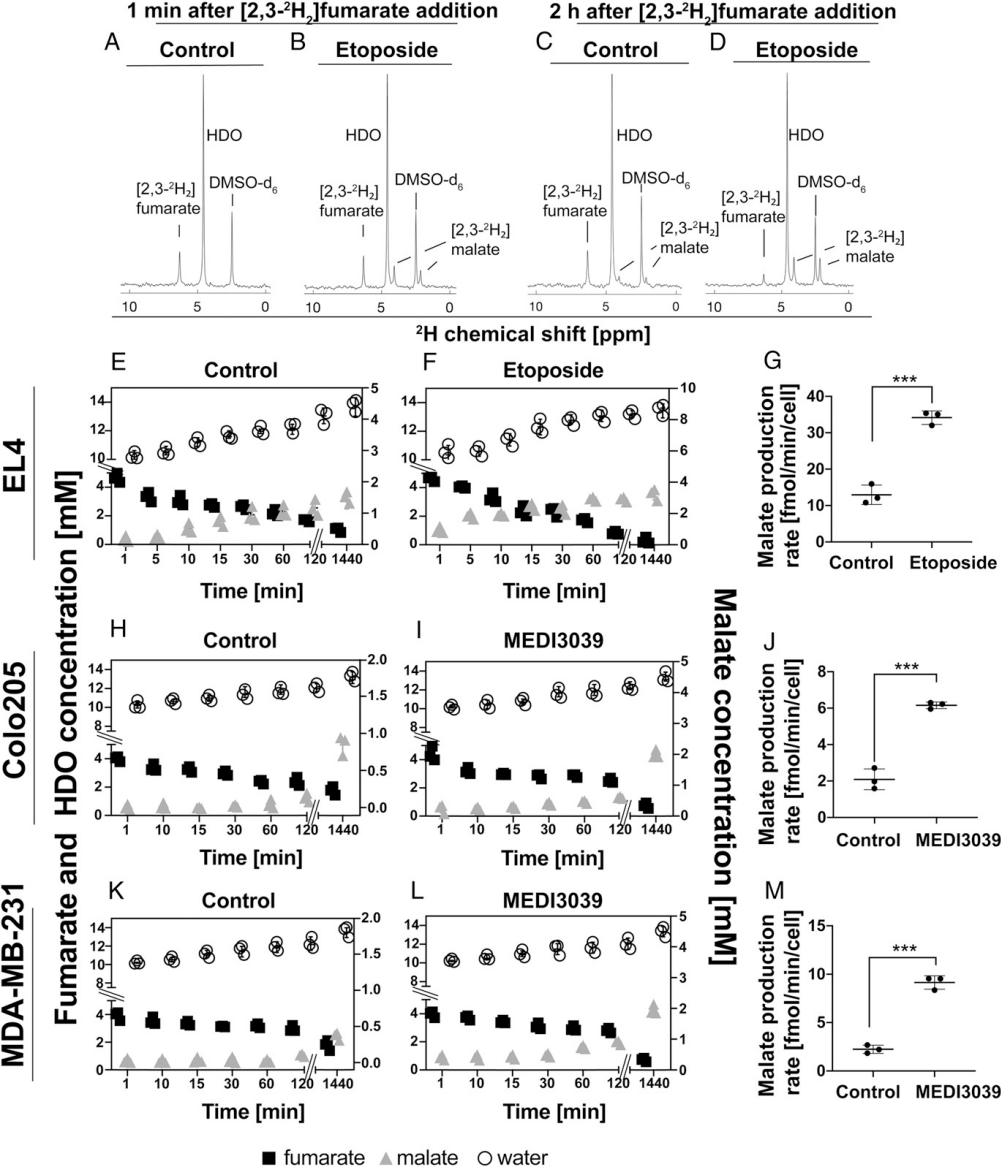
(*A*–*D*) ^2^H NMR spectra of murine lymphoma (EL4) cell culture medium. (*A*) Medium from untreated cells and (*B*) cells treated for 24 h with 15 μM etoposide, 1 min after the addition of 5 mM [2,3-^2^H_2_]fumarate. (*C*) Medium from untreated cells and (*D*) cells treated for 24 h with etoposide, 2 h after the addition of [2,3-^2^H_2_]fumarate. (*E*, *F*) Deuterated fumarate, malate, and water concentrations in medium from untreated (*E*) and etoposide-treated cells at the indicated times after addition of 5 mM [2,3-^2^H_2_]fumarate (*F*). Rate of malate production in untreated and etoposide-treated EL4 cell suspensions (**P = 0.0018) (±SD, *n* = 3 biological replicates) (*G*). (*H*–*M*) Production of labeled malate and water in cultures of human colorectal (Colo205) and breast cancer (MDA-MB-231) cells following the addition of 5 mM [2,3-^2^H_2_]fumarate. Deuterated fumarate, malate, and water concentrations in medium from untreated (*H*, *K*) and cells treated for 24 h with MEDI3039 (*I*, *L*), at the indicated times after the addition of 5 mM [2,3-^2^H_2_]fumarate. (*J*, *M*) Rate of labeled malate production in untreated and MEDI3039-treated Colo205 (***P = 0.008) (*J*) and MDA-MB-231 (***P < 0.0001) cells. (*M*) (Data are presented as mean ± SD, *n* = 3 biological replicates).

Cultured murine lymphoma cells (EL4) (Fig. 1) were treated with 15 μM etoposide or drug vehicle (control) for 24 h. Cell viability was 90 ± 3% prior to treatment and 9 ± 4% at 24 h post etoposide treatment. Five mM [2,3-^2^H_2_]fumarate was added to the cell suspensions, and samples were taken for ^2^H NMR measurements of the deuterated fumarate, malate, and water concentrations. As early as 1 min after [2,3-^2^H_2_]fumarate addition, etoposide-treated cells showed increased malate production (Fig. 1 *A* and *B*). The deuterium-labeled malate concentration was 1.3 ± 0.4 mM in drug-treated cell suspensions as compared to 0.17 ± 0.04 mM in controls (*P* < 0.0001, *n* = 3 per group). The labeled malate production rate was significantly higher (*P* = 0.0004) in the etoposide-treated group (34.1 ± 1.8 fmol/min/cell) compared to controls (12.9 ± 2.7 fmol/min/cell) (Fig. 1*G*). Similar results were obtained in colorectal (Colo205) (Fig. 1 *H*–*J*) and triple negative breast cancer (MDA-MB-231) cells (Fig. 1 *K*–*M*). The rates of malate production posttreatment were higher in Colo205 cells (6.1 ± 0.2 fmol/min/cell) (Fig. 1*J*) and MDA-MB-231 cells (9.1 ± 0.7 fmol/min/cell) (Fig. 1*M*) compared to untreated controls (Colo205, 2.1 ± 0.6 fmol/min/cell; MDA-MB-231, 2.2 ± 0.4 fmol/min/cell). These rates were much lower than those observed in treated EL4 cells; however, there was much less cell death, with the viabilities of these two cell lines posttreatment being much higher (Colo205, 56 ± 2.4%, MDA-MB-231, 55 ± 4.1%) than in EL4 cells (9 ± 4%). The rates of water labeling, which can be explained by tricarboxylic acid (TCA) cycle activity (17, 18, 22, 23), were not significantly affected by treatment. The rate in untreated EL4 cells was 30.1 ± 2.4 fmol/min/cell, in MDA-MB-231 cells was 20.3 ± 4.9 fmol/min/cell, and in Colo205 cells was 16.7 ± 5.8 fmol/min/cell, whereas in treated cells, the corresponding rates were 35.6 ± 1.2, 18.7 ± 4.9, and 19.3 ± 2.4 fmol/min/cell, respectively, indicating that there was retention in the necrotic cells of the activities of those enzymes responsible for exchange of fumarate deuterons with water. Moreover, these rates were similar to the rates of fumarate utilization, which in untreated EL4 cells was 31.9 ± 3.8 fmol/min/cell, in MDA-MB-231 cells was 10.1 ± 5.1 fmol/min/cell, in Colo205 cells was 17.6 ± 3.1 fmol/min/cell, and in the treated cells was 38.9 ± 2.4, 12.3 ± 3.9, and 20.7 ± 6.9 fmol/min/cell, respectively.

### Deuterated Fumarate Metabolism Detects Tumor Cell Death In Vivo.

**Fig. 2.**
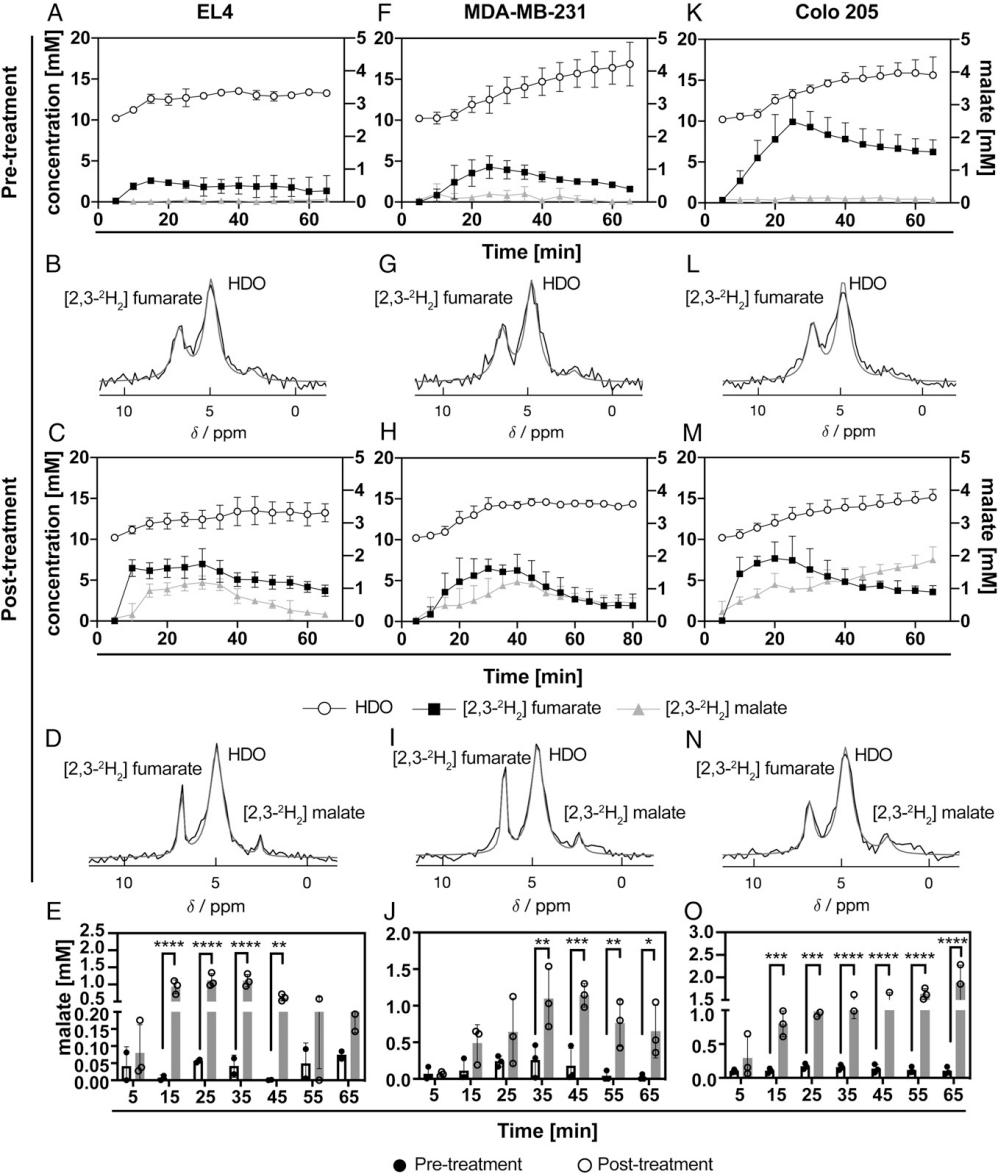
^2^H MR spectroscopic measurements of labeled fumarate, malate, and water concentrations in EL4 (*A*–*E*), MDA-MB-231 (*F*–*J*), and Colo205 (*K*–*O*) tumors. Tumor spectra were acquired before and 48 h after etoposide treatment (67 mg/kg) of EL4 tumor-bearing mice (*n* = 3, **P < 0.01, ****P < 0.0001) and before and 24 h after treatment of MDA-MB-231 (*n* = 5, *P < 0.05, **P < 0.01, ***P < 0.001) and Colo205 tumor-bearing mice (*n* = 5, ***P < 0.001, ****P < 0.0001) with MEDI3039 (0.8 mg/kg). (*B*, *D*, *G*, *I*, *L*, *N*) Sum of 12 ^2^H spectra recorded over 60 min. The [2,3-^2^H_2_] fumarate injection (1 g/kg) started 5 min after the start of acquisition of the first spectrum. The peaks were fitted individually using prior knowledge. (*E*, *J*, *O*) Estimated tumor malate concentrations pre- and posttreatment at indicated time points after [2,3-^2^H_2_] fumarate injection. (*P < 0.05, **P < 0.01, ***P < 0.001, ****P < 0.0001). Data are shown as mean ± SD.

**Fig. 3.**
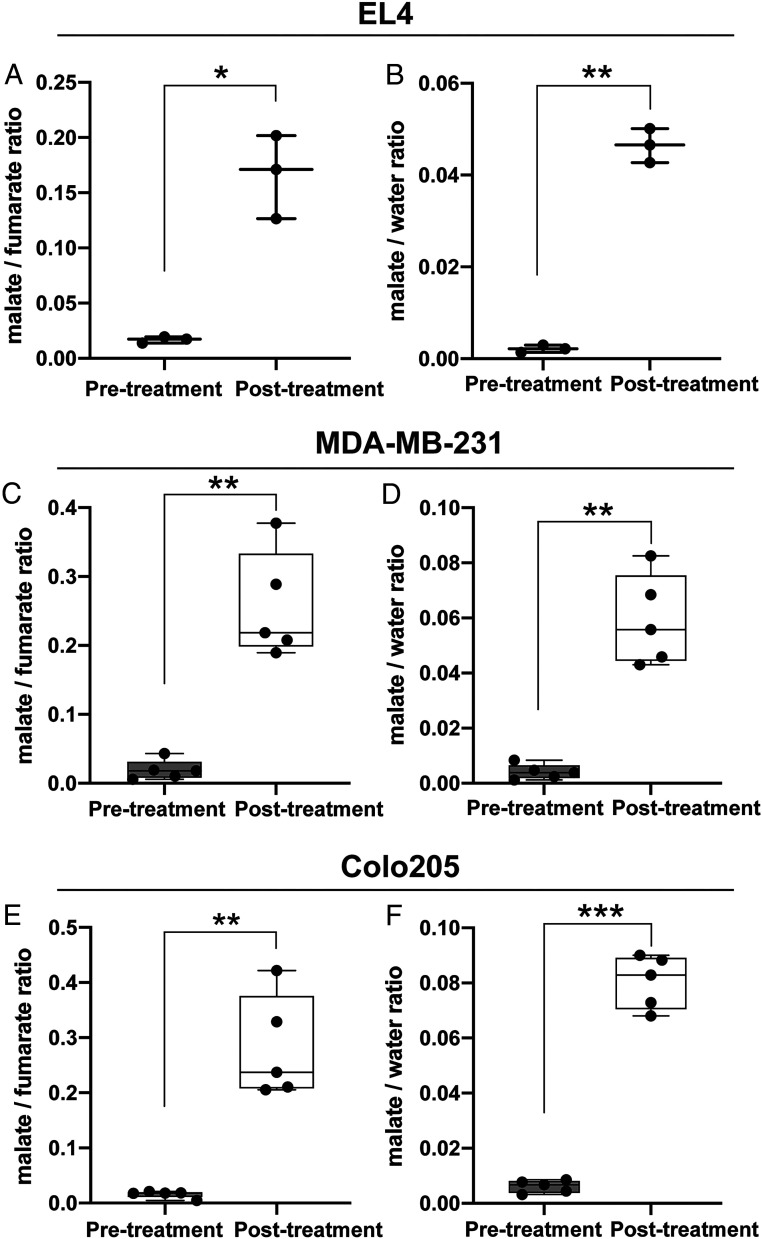
Malate/fumarate and malate/water signal ratios in EL4, MDA-MB-231, and Colo205 tumors before and after treatment. (*A*) Tumor [2,3-^2^H_2_]malate/[2,3-^2^H_2_]fumarate and (*B*) [2,3-^2^H_2_]malate/semiheavy water (HDO) ratios, obtained by summing the fumarate, malate, and HOD signals over 65 min after injection of [2,3-^2^H_2_]fumarate, before treatment, and at 48 h after treatment of EL4 tumor-bearing mice with etoposide (67 mg/kg) (*n* = 3, *P < 0.05, **P < 0.01). Data are presented as mean ± SD (*C*) [2,3-^2^H_2_]malate/[2,3-^2^H_2_]fumarate and (*D*) [2,3-^2^H_2_]malate/semiheavy water (HDO) ratios before and at 24 h after treatment of MDA-MB-231 tumor-bearing mice with MEDI3039 (0.8 mg/kg) (*n* = 5, **P < 0.01). (*E*) [2,3-^2^H_2_]malate/[2,3-^2^H_2_]fumarate ratio and (*F*) [2,3-^2^H_2_]malate/semiheavy water (HDO) ratios before and at 24 h after treatment of Colo205 tumor-bearing mice with MEDI3039 (0.8 mg/kg) (*n* = 5, **P < 0.01, ***P < 0.001). Data are shown as box and whisker plots.

Deuterium-labeled fumarate, malate, and water concentrations were monitored in EL4, MDA-MB-231, and Colo205 tumors using localized ^2^H spectroscopy measurements following intravenous injection of 1 g/kg [2,3-^2^H_2_]-disodium fumarate (Fig. 2). In all three implanted tumor models, there was a significant increase in labeled malate concentration following drug treatment, which in the EL4 tumors was confirmed by measurements of the concentrations in freeze-clamped tumor extracts (Table 1). In untreated tumors, the concentration of [2,3-^2^H_2_]malate, determined by ^2^H NMR, was only 10% of the unlabeled concentration, determined by ^1^H NMR, whereas in treated tumors the total malate concentration increased ∼7×, of which ∼30% was deuterium labeled (Table 1). In both the untreated and treated tumors, the fumarate was ∼50% labeled, indicating that there had been substantial solvent exchange of fumarate deuterons. The tumor malate/fumarate and malate/HDO signal ratios measured in vivo at 48 h (EL4) or 24 h (MDA-MB-231 and Colo205) after treatment are shown in Fig. 3.

**Table 1.** Deuterium-labeled fumarate, malate, and water concentrations measured in tissue extracts using ^2^H NMR

		Control		Etoposide-treated	
		Concentrations of deuterated species			
	HDO (µmols/g)	[2,3-^2^H_2_]fumarate (µmols/g)	[2,3-^2^H_2_]malate (µmols/g)	[2,3-^2^H_2_]fumarate (µmols/g)	[2,3-^2^H_2_]malate (µmols/g)
Tumor		5.23 ± 0.63	0.08 ± 0.02	6.82 ± 0.84	1.81 ± 0.53
Kidney		11.03 ± 1.01	0.58 ± 0.03	7.20 ± 0.82	1.27 ± 0.39
Liver		2.06 ± 0.25	not detected (n.d.)	2.14 ± 0.52	n.d.
Muscle		0.017 ± 0.011	n.d.	0.050 ± 0.016	n.d.
Heart					
0	18.80 ± 0.50				
20 min	20.48 ± 0.69	1.17 ± 0.25	0.27 ± 0.18		
60 min	21,21 ± 0.78	0.87 ± 0.024	0.31 ± 0.09		
		Concentrations of protonated species			
Tumor		Fumarate (µmols/g)	Malate (µmols/g)	Fumarate (µmols/g)	Malate (µmols/g)
		6.92 ± 2.65	0.75 ± 0.44	7.72 ± 1.72	3.74 ± 0.21

The concentrations of the protonated species in tumors were measured using ^1^H NMR. For malate, this was based on the upfield ^2^H and ^1^H resonances at 2.4 ppm since the downfield resonance was not resolved from the water resonance. The concentrations were measured in the indicated tissues 20 min after intravenous injection of [2,3-^2^H_2_]fumarate. The measurements in the tumor, kidney, liver, and skeletal muscle were made in EL4 tumor-bearing mice, which were either etoposide- (67 mg/kg) or vehicle-treated (control). The measurements in the heart muscle were made in nontumor-bearing mice, and extracts were made before (0) and at the indicated times after [2,3-^2^H_2_]fumarate injection. Data are expressed as the mean ± SEM (*n* = 3).

The labeled malate concentration in etoposide-treated EL4 tumors (Fig. 2 and Table 1) was ∼4× the labeled malate concentration in blood (Table 2). Moreover, the labeled malate concentration in blood increased markedly posttreatment, consistent with the treated tumor being the major source of labeled blood malate. Labeled malate was undetectable in the liver and muscle but present in the kidney at much higher concentration than in the blood, both before and after treatment. The increase in malate concentration in the kidney posttreatment was comparable with the increase in the blood concentration. The labeled fumarate concentration in the kidney pretreatment was similar to the blood concentration and ∼2× that in the tumor and 5× that in the liver. There were only very low concentrations of labeled fumarate in skeletal muscle. Heart muscle takes up fumarate rapidly (24), and we observed labeled malate concentrations in this tissue that exceeded the blood concentration in untreated animals (Tables 1 and 2). The labeled water concentration in heart muscle was similar to that in the blood pool. Erythrocytes will also take up fumarate and produce malate on the time scale of the experiments shown here (25). Incubation of erythrocytes with [2,3-^2^H_2_]fumarate produced concentrations of labeled malate comparable to those observed in the heart. However, there was no increase in water labeling (Table 3), reflecting the low levels of TCA cycle enzymes in these cells (26).

**Table 2.** Deuterium-labeled fumarate and malate concentrations in the blood of EL4 tumor-bearing mice before (BT) and after (AT) etoposide treatment

	Concentration at specified time points following injection of [2,3-^2^H_2_]fumarate (mM)			
	20 min		70 min	
Metabolite	BT	AT	BT	AT
[2,3-^2^H_2_]fumarate	12.35 ± 3.61	10.08 ± 0.89	4.95 ± 1.43	4.45 ± 0.50
[2,3-^2^H_2_]malate	0.03 ± 0.01	0.47 ± 0.05	0.004 ± 0.003	0.19 ± 0.09
HDO	20.16 ± 1.14	19.32 ± 1.68	22.68 ± 1.42	23.52 ± 1.21

Blood was collected by cardiac puncture at the specified times after intravenous injection of 1 g/kg [2,3-^2^H_2_]fumarate and the concentrations measured using ^2^H NMR. Data are expressed as mean ± SEM (*n* = 3).

**Table 3.** Deuterium-labeled fumarate, malate, and water concentrations measured in mouse erythrocyte suspensions using ^2^H NMR

Time after [2,3-^2^H_2_]fumarate addition (min)	[2,3-^2^H_2_]fumarate [mM]	[2,3-^2^H_2_]malate [mM]	HDO [mM]
5	7.45 ± 0.39	0.04 ± 0.03	11.73 ± 0.28
60	5.35 ± 0.89	0.25 ± 0.05	11.91 ± 0.50

The concentrations were measured at the indicated time points after the addition of 8 mM [2,3-^2^H_2_]fumarate. Data are expressed as mean ± SEM (*n* = 3).

The tumor fumarate signal in the EL4 and MDA-MB-231 tumor models increased significantly following drug treatment (Fig. 2 *A*–*D*), implying that there was increased perfusion since the blood fumarate concentration did not change significantly following etoposide treatment (Table 2). To confirm changes in tumor perfusion, dynamic contrast enhanced (DCE)-MRI measurements were performed in EL4, MDA-MB-231, and Colo205 tumor-bearing mice.

### DCE-MRI Showed an Increase in Perfusion of EL4 and MDA-MB-231 Tumors after Treatment.

In separate cohorts of MDA-MB-231 (*n* = 3), EL4 (*n* = 3), and Colo205 (*n* = 3) tumor-bearing mice, DCE-MRI was used to assess tumor perfusion following drug treatment (*SI Appendix*, Fig. S1). Tumor gadolinium (Gd^3+^) concentrations pre- (0.25 ± 0.02 mM) and posttreatment (0.27 ± 0.016 mM) showed no difference (*P* = 0.9953) in the Colo205 tumor model (*SI Appendix*, Fig. S1*B*) but were increased slightly by treatment in MDA-MB-231 and EL4 tumor-bearing mice (*SI Appendix*, Fig. S1 *D* and *F*), increasing from 0.19 ± 0.05 to 0.39 ± 0.09 mM (*P* = 0.0021) and 0.18 ± 0.01 to 0.59 ± 0.08 (*P* = 0.0001), respectively, at ∼10 min after intravenous injection of the contrast agent.

### Fumarase Activity Is Decreased in Cells and Tumors by Drug Treatment.

**Fig. 4.**
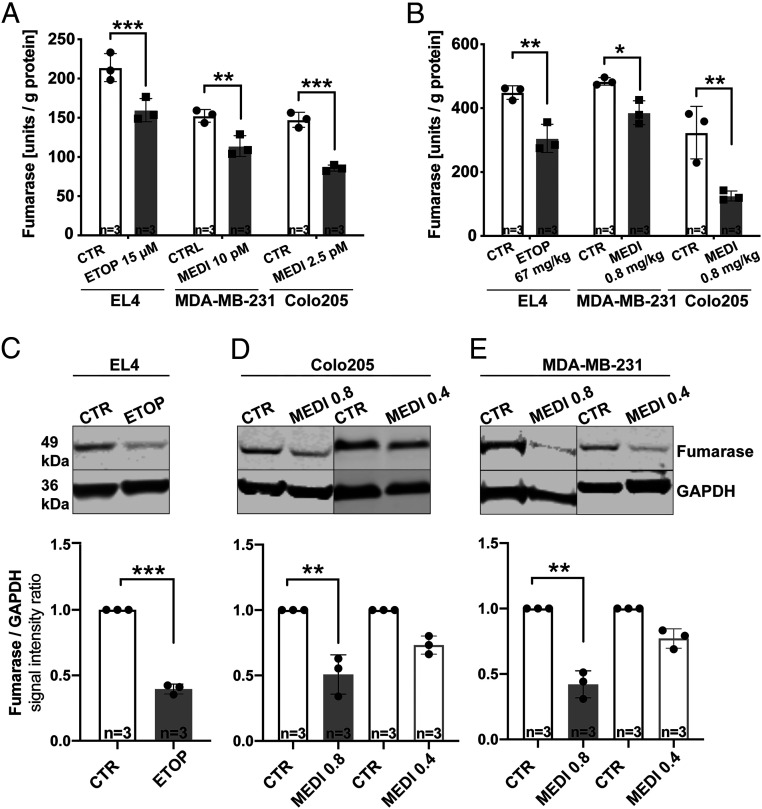
Fumarase activity in the EL4, Colo205, and MDA-MB-231 cells (*A*) and tumors (*B*) following treatment with the indicated drug concentrations. Western blot analysis of fumarase expression in EL4 (*C*), Colo205 (*D*), and MDA-MB-231 (*E*) tumors following treatment with the indicated drug concentrations. Densitometric analysis of the Western blots. Fumarase band intensities relative to those of GAPDH (three biological replicates, data are presented as mean ± SEM, *P < 0.05, **P < 0.01, ***P < 0.001).

Increased conversion of fumarate to malate has been attributed to compromised plasma membrane integrity in necrotic cells and consequently increased access of fumarate to the enzyme (9). To confirm this, we measured fumarase activity in drug-treated cells in culture and in drug-treated tumors in vivo (Fig. 4). Enzyme activity was decreased in cells, and enzyme activity and protein levels were decreased in tumors, by drug treatment, confirming loss of plasma membrane integrity and leakage of the enzyme from the cell (9, 15).

### Confirmation of Cell Death in Drug-Treated Tumors.

**Fig. 5.**
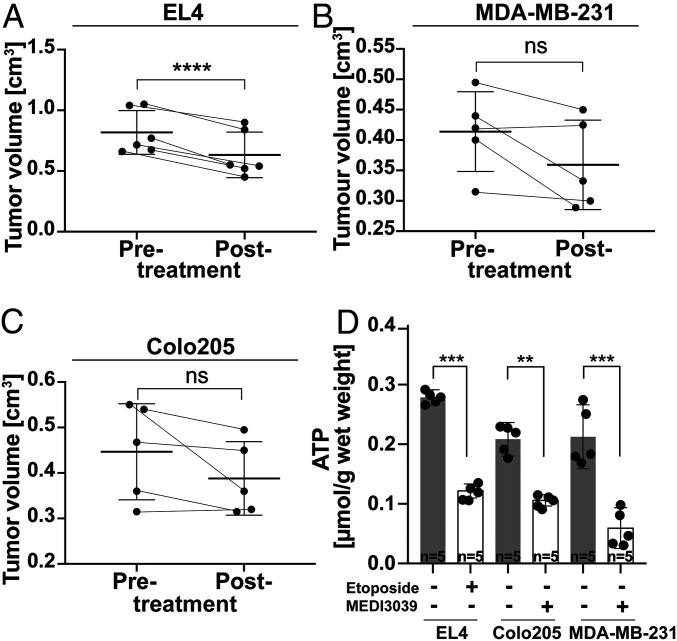
Volumes of (*A*) EL4, (*B*) MDA-MB-231, and (*C*) Colo205 tumors before and 48 h after etoposide treatment (EL4) or 24 h after MEDI3039 treatment (MDA-MB-231, Colo205) (*n* = 5, data are presented as mean ± SD, ****P < 0.0001) (*D*) ATP concentrations measured in tumor extracts before (*n* = 5) and after (*n* = 5) treatment; data are presented as mean ± SD, **P < 0.01, ***P < 0.001.

**Fig. 6.**
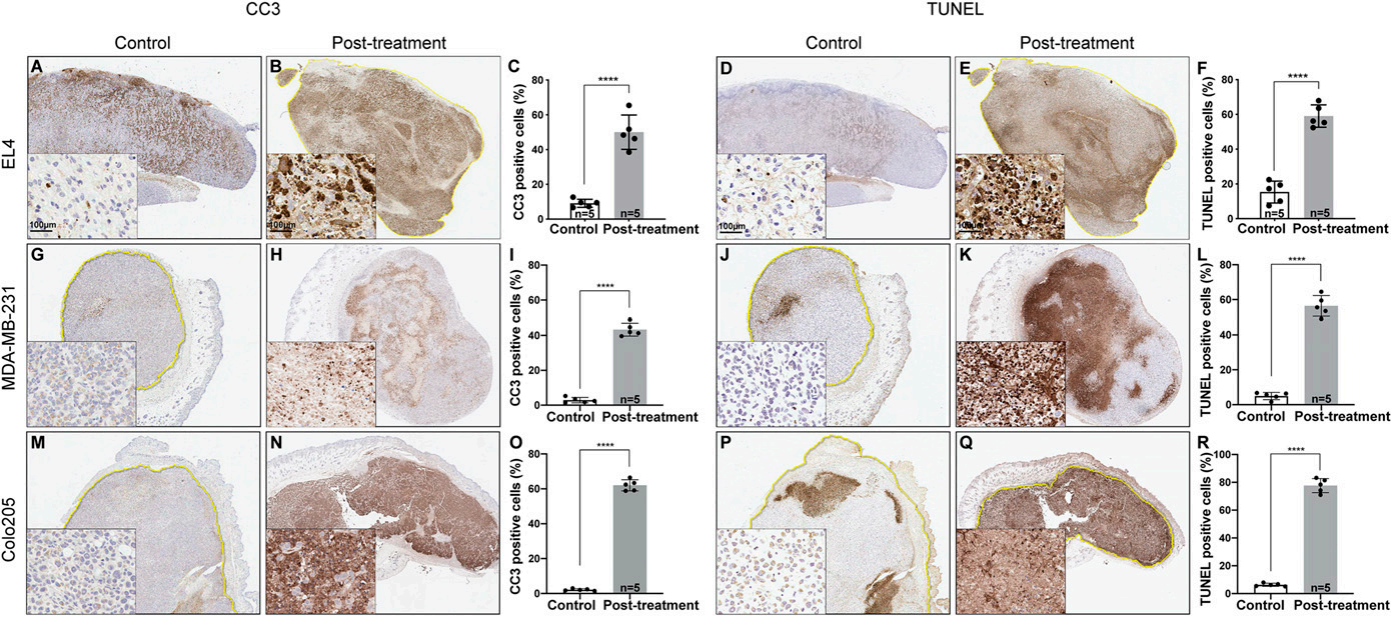
Histological assessment of tumor cell death following treatment. Tumor sections were stained for CC3 and TUNEL (*n* = 5 per group, drug- and vehicle-treated). CC3 (*A*, *B, C*) and TUNEL (*D*, *E, F*) staining of EL4 tumor sections taken 48 h after treatment of the animals with etoposide or drug vehicle (control). CC3 (*G*, *H*, *I*, *M*, *N,* *O*) and TUNEL (*J*, *K*, *L*, *P*, *Q, R*) staining of MDA-MB-231 and Colo205 tumor sections taken 24 h after treatment of the animals with MEDI3039 (0.8 mg/kg) or drug vehicle (control). ****P < 0.0001.

Cell death in drug-treated tumors was confirmed by measuring tumor size and ATP concentrations (Fig. 5) and by histological assessment of the levels of cleaved capase 3 (CC3) and DNA damage (TUNEL) (Fig. 6). Although there was no significant decrease in the size of MDA-MB-231 and Colo205 tumors after MEDI3039 treatment, there were nevertheless significant decreases in tumor ATP content and increases in CC3 and TUNEL staining, as we have observed previously in these tumor models following MEDI3039 treatment (7). EL4 tumors showed a significant decrease in volume, from 0.7 ± 0.3 cm^3^ to 0.5 ± 0.1 cm^3^ at 48 h after etoposide treatment, and increases in CC3 and TUNEL staining.

### ^2^H Imaging of Cell Death Using [2,3-^2^H_2_]fumarate.

**Fig. 7.**
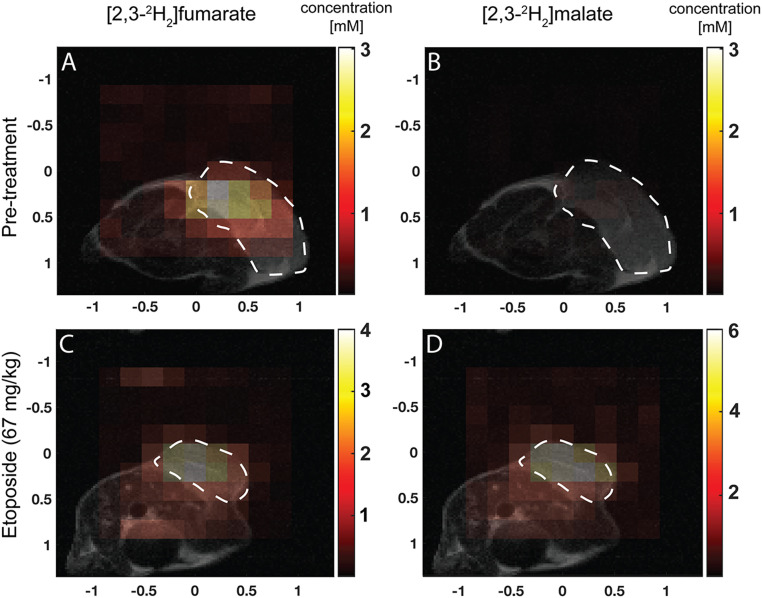
Metabolite concentration maps in the central slice derived from dynamic three-dimensional CSI images summed over the first 30 min of signal acquisition following [2,3-^2^H_2_]fumarate injection into EL4 tumor-bearing mice. The color code represents concentration (in mM) derived from the ratios of the peak intensities in the malate and fumarate maps to peak intensities in an initial HDO map and corrected for the number of ^2^H labels per molecule and signal saturation. (*A*–*D*) The locations of the tumors are outlined by dotted white lines. Approximate concentration maps of (*A*) fumarate pretreatment; (*B*) malate pretreatment; (*C*) fumarate 48 h posttreatment; (*D*) malate 48 h posttreatment.

A fast, dynamic three-dimensional chemical shift imaging sequence was used to capture spatial information about the conversion of [2,3-^2^H_2_]fumarate to [2,3-^2^H_2_]malate in implanted EL4 tumors in vivo following a bolus injection of labeled fumarate (1 g/kg) into tumor-bearing mice, both pre- and posttreatment (Fig. 7). No malate signal was detected pretreatment, whereas significant malate production was observed 48 h after etoposide treatment. The signal was localized to the tumor area. The fumarate concentration also increased in the tumor posttreatment in agreement with the DCE MRI, tissue extract, and spectroscopy data.

## Discussion

^13^C MR spectroscopy and spectroscopic imaging of [1,4-^13^C_2_]malate production from hyperpolarized [1,4-^13^C_2_]fumarate has been used previously to detect early evidence of tumor cell death in EL4 tumors following treatment with etoposide (9) and in MDA-MB-231 tumors following treatment with doxorubicin (10). Production of labeled malate by necrotic cells has been attributed to a compromised plasma membrane permeability barrier allowing fumarate to rapidly gain access to cell fumarase with consequent conversion to malate within the short lifetime of the ^13^C spin polarization, thus providing a positive marker of tumor cell necrosis. We have shown here, using deuterium labeled fumarate, that the limited lifetime of the ^13^C spin polarization, which is of the order of 1 to 2 min in vivo (27, 28), is not a prerequisite for an effective cell death measurement using labeled fumarate. Untreated tumors had only low levels of [2,3-^2^H_2_]malate at 60 min after injection of [2,3-^2^H_2_]fumarate, whereas in treated tumors elevated levels of [2,3-^2^H_2_]malate were detected within 15 min. Increased malate production in dead or dying cells cannot be explained by increased fumarase activity. As was observed previously in studies with hyperpolarized [1,4-^13^C_2_]fumarate (9), enzyme activity decreased in drug-treated cells and tumors reflecting leakage of the enzyme from necrotic cells into the cell culture medium and tumor interstitial space. Increased malate production can also not be explained by increased delivery of labeled fumarate to the tumor posttreatment. Although the EL4 and MDA-MB-231 tumors both showed significantly higher levels of [2,3-^2^H]fumarate posttreatment, which DCE-MRI measurements showed was due to increased perfusion, this is unlikely to be responsible for the increased [2,3-^2^H_2_]malate signal posttreatment since the fumarate concentration in these tumors, both pre- and posttreatment, was in considerable excess of the *K*_m_ of fumarase for fumarate, which has been measured at 5 μM (29). Moreover, Colo205 tumors showed a similar increase in the levels of labeled malate posttreatment with no change in tumor perfusion, as assessed from the concentrations of [2,3-^2^H_2_]fumarate and gadolinium contrast agent. Furthermore, detecting cell death through an increase in the malate/fumarate signal ratio corrects for the effects of any changes in perfusion since the fumarate signal provides a measure of this.

While fumarate was taken up by viable cells, as evidenced by water labeling observed in the cell experiments, there was no subsequent accumulation of labeled malate, which we only observed in drug-treated cells and tumors. Deuterons can be lost from [2,3-^2^H_2_]fumarate in the sequential reactions catalyzed by fumarase and malate dehydrogenase and subsequent keto-*enol* tautomerization of the oxaloacetate produced (30). The similarity between the rate of fumarate consumption and water labeling in all three cell lines suggests that fumarate is taken up by viable cells and metabolized to malate and subsequently to oxaloacetate. The absence of a significant increase in the rate of water labeling in Colo205 and MDA-MB-231 cells following drug treatment, when >40% of the cells had become necrotic, suggests that in these cells the rate is limited by malate dehydrogenase activity and the rate of oxaloacetate tautomerization. The rates of fumarate utilization increased in all three cell lines following treatment, consistent with the proposal that the increase in malate labeling is due to increased access of fumarate to fumarase in necrotic cells. However, there was no concordance between the increase in the rates of fumarate utilization and malate labeling posttreatment across the three cell lines (7 versus 21 fmols/min/cell, respectively, in EL4 cells, 3 versus 4 fmols/min/cell in Colo205 cells, and 2 versus 7 fmols/min/cell in MDA-MB-231 cells), suggesting that the observation of increased malate labeling may also be due to an increase in the steady state level of malate in drug-treated cells resulting from reduced consumption in the TCA cycle. ^2^H and ^1^H NMR measurements on EL4 tumor extracts showed a large increase in the concentrations of labeled and unlabeled malate posttreatment. In untreated tumors only ∼10% of the malate was deuterium labeled, which is likely due to production of malate from other carbon sources and also exchange of deuterium label in malate with solvent water in the reversible reaction catalyzed by malate dehydrogenase and subsequent keto-*enol* tautomerization of oxaloacetate. Following etoposide treatment, the total malate concentration increased 7×, of which 30% was deuterium labeled. Only ∼50% of the fumarate in the tumors was deuterium labeled, showing that there had been substantial solvent exchange in the tumor and other body tissues.

Measurements in tissue extracts showed high levels of labeled fumarate in the kidneys (11 μmols/g), presumably reflecting rapid renal excretion, as we have observed previously with hyperpolarized [1,4-^13^C_2_]fumarate (15), with lower levels in the tumor and lower levels still in the liver and heart (Table 1). Labeled fumarate in the liver and heart muscle can be explained, at least in part, by labeled fumarate present in the blood pool. With a blood [2,3-^2^H_2_]fumarate concentration of 12 mM at 20 min after injection (Table 2), and using blood volumes in mouse liver and kidney of ∼0.2 mL/g tissue (31), and accessible volumes (vascular plus interstitial) in liver and heart muscle ∼0.2 mL/g (32) gives a calculated concentration of labeled fumarate in the liver, kidney, and heart muscle blood pools of ∼2.4 μmols/g tissue. This is similar to the measured tissue concentration of 2.1 μmols/g in liver, although above that measured in the heart, which was 1.2 μmols/g. The evidence that fumarate is largely present in the liver blood pool and not in the liver cells is further supported by the absence of detectable [2,3-^2^H_2_]malate in the liver. The highest malate concentration in the blood would contribute only ∼0.1 μmols/g liver and was at the limit of detection in these ^2^H NMR measurements on liver extracts (Table 1). However, the labeled malate concentration in the heart muscle was 0.3 μmols/g, 10× that in the blood and indicating substantial uptake and metabolism of fumarate in the heart muscle, as has been observed previously (24). This may explain why the fumarate concentration in the heart was lower than that expected based on the concentration in the blood pool. Fumarate uptake and metabolism in the heart may also have contributed to the observed water labeling in this tissue. However, since the labeled water concentration was similar to that in the blood, we cannot distinguish between labeled water generated in the heart and that washed in from other tissues. The labeled malate concentration in the kidney at 20 min after labeled fumarate injection (0.6 μmols/g) was much higher than can be accounted for by the [2,3-^2^H_2_]malate concentration in the blood (0.03 mM). Some of this labeled malate may have been produced in the kidney but some may have been produced elsewhere in the body and was undergoing excretion via the kidneys. In etoposide-treated EL4 tumor-bearing mice, the kidney malate concentration more than doubled to 1.3 μmols/g. Although the blood concentration also increased, to 0.5 mM, again the blood pool can make only a very small contribution to the total kidney malate concentration. Some of this malate is presumably malate that has washed out of the treated tumor, where the labeled malate concentration increased from 0.08 μmols/g prior to treatment to 1.8 μmols/g posttreatment, at 20 min after injection of [2,3-^2^H_2_]fumarate. Importantly the malate concentration in the tumor at this time point was ∼4× higher than the blood concentration, demonstrating that the malate had been produced by cell death in the tumor rather than being washed in from elsewhere in the body.

The production of malate from fumarate makes fumarate a positive contrast agent for detecting cell death and in principle should be more sensitive for detecting cell death than imaging techniques that rely on a decrease in signal, for example, a decrease in tumor lactate labeling in animals injected with hyperpolarized [1-^13^C]pyruvate (5, 7). In the same Colo205 and MDA-MB-231 tumor models that were used here, we have shown previously that treatment with MEDI3039 resulted in a decrease in lactate labeling from hyperpolarized [1-^13^C]pyruvate (expressed as the ^13^C lactate/pyruvate signal ratio) of 42.2 ± 15.9% and 36.3 ± 18.6% in Colo205 and MDA-MB-231 tumors, respectively (7). There was an increase in TUNEL staining in the Colo205 tumors, from 8.0 ± 6.7% to 19.4 ± 6.3% and in the MDA-MB-231 tumors from 6.6 ± 2.0% to 21.1 ± 6.1%. Expressing the percentage decrease in lactate labeling as a ratio of the increase in the percentage of cell death gives values of 3.7 and 2.5 for Colo205 and MDA-MB-231 tumors, respectively. In the study presented here, MEDI3039 treatment resulted in an increase in the [2,3-^2^H_2_]malate/[2,3-^2^H_2_]fumarate signal ratio from 0.016 ± 0.04 to 0.28 ± 0.26 in Colo205 tumors, a 1650% increase, and an increase from 0.019 ± 0.03 to 0.25 ± 0.23 in MDA-MB-231 tumors, an increase of 1216%. TUNEL staining increased from 6.3 ± 2.3% to 77.6 ± 5.6% in Colo205 tumors and from 5.0 ± 1.7% to 56.4 ± 3.9% in MDA-MB-231 tumors. Expressing the percentage increase in malate labeling as a ratio of the increase in the percentage of cell death gives values of 23 and 24 for Colo205 and MDA-MB-231 tumors, respectively. Therefore, monitoring malate production from labeled fumarate is indeed a more sensitive method for detecting cell death than monitoring the decrease in lactate labeling from hyperpolarized [1-^13^C]pyruvate. This assessment of sensitivity, however, does not take into account the much lower signal-to-noise ratio (SNR) in the ^2^H spectra, which also has a large effect on our estimate of the sensitivity of the deuterated fumarate experiment for detecting cell death given the very low intensity of the malate ^2^H signal in the untreated tumors. The SNRs of the most intense malate peak in the ^2^H spectra of treated Colo205 and MDA-MB-231 tumors were 1.7 ± 0.6 and 2.3 ± 0.9, respectively, whereas the lactate SNRs in the ^13^C spectra of these tumors published previously were 44 ± 7 (7) and 46 ± 8, respectively (10). The differences in SNR between the ^2^H and ^13^C experiments will also depend on coil configuration and performance.

We have shown previously that detecting cell death with hyperpolarized [1,4-^13^C]fumarate is more effective than diffusion-weighted ^1^H MRI at detecting low levels of diffuse necrosis (11). Treatment of EL4 tumors with an anti-vascular drug that resulted in small areas of necrosis at 6 h after treatment resulted in a significant increase in labeled malate production with no change in the apparent diffusion coefficient (ADC) of tissue water in a diffusion-weighted imaging experiment. This was consistent with a previous study, which showed in tumors with small and diffuse regions of necrosis that there can be no change in ADC, even with necrotic fractions of up to 40% (33). Malate detection effectively integrates cell death over the whole imaging voxel, since it is a positive contrast agent, whereas with small and diffuse regions of necrosis in the image voxel, detection of cell death using diffusion-weighted MRI involves measuring relatively small increases in the water ADC. Assessing cell death in vivo using [2,3-^2^H_2_]fumarate is simpler than with hyperpolarized [1,4-^13^C]fumarate since the method does not require a hyperpolarizer and as a result is also less expensive to implement. However, it does require much higher concentrations of labeled fumarate; 0.4 mL of 312 mM [2,3-^2^H_2_]fumarate was used here as compared to 0.2 mL of 20 mM hyperpolarized [1,4-^13^C]fumarate used previously (9). Despite the higher fumarate concentration, the SNR of the most intense [2,3-^2^H]malate signal was only 2.9 ± 0.2 in the spectra of etoposide-treated EL4 tumors, which were the sum of 2,142 transients acquired over 5 min using a 67° pulse and a repetition time of 0.14 s and 4.5 ± 0.5 in the sum of the 13 spectra acquired over 65 min. Signal was acquired from the sensitive volume of the surface coil, which imaging experiments showed included mainly tumor tissue. Contrast this with ^13^C spectroscopy measurements of hyperpolarized [1,4-^13^C]fumarate metabolism in etoposide-treated EL4 tumors where the malate SNR in spectra, which were the sum of 60 transients acquired over 3 min using a 10° pulse, a repetition time 3 s, and a slice thickness of 5 mm, was 17 (9). Increasing the concentration of [2,3-^2^H_2_]fumarate further was not possible as this started to affect breathing and heart rates, which may have been a consequence of the sodium ion present in the preparation. The lethal dose 50% (LD_50_) for intravenous sodium chloride in mice is 645 mg/kg, which is ∼2.8 osmol/L; we injected 1 osmol/L disodium fumarate. However, ^2^H MRS measurements of [2,3-^2^H]malate production from [2,3-^2^H]fumarate appear to be an intrinsically more sensitive method for detecting cell death than ^13^C MRS measurements of [1,4-^13^C]malate production from hyperpolarized [1,4-^13^C]fumarate. In EL4 tumor-bearing mice injected with hyperpolarized [1,4-^13^C]fumarate, the rate constant describing labeled malate production in the tumor increased by a factor of 2.4 following etoposide treatment, and the ratio of the areas under the malate and fumarate labeling curves increased by a factor of 1.6, where this was the result of an increase in tumor cell necrosis from ∼5% to ∼30% (9). Whereas in this study, in EL4 tumor-bearing mice injected with [2,3-^2^H_2_]fumarate, the ratio of the areas under the malate and fumarate labeling curves increased by a factor of 10, increasing from 0.016 ± 0.02 to 0.16 ± 0.14 (*P* = 0.0024, *n* = 3) following etoposide treatment, with the estimated levels of tumor cell necrosis increasing from 15 ± 5% to 59 ± 6%. This greater sensitivity of the ^2^H experiment for detecting cell death can be explained by the extended period over which the accumulation of labeled malate is measured, 65 mins, as compared to just 3 min in the case of the hyperpolarized ^13^C experiment, which is limited by the short lifetime of the ^13^C hyperpolarization.

The chemical shift separation of the fumarate, water, and malate resonances and the concentrations of fumarate and malate observed in the tumors suggest that this technique for detecting tumor cell death posttreatment could translate to the clinic. De Feyter et al. (17) observed partially resolved resonances from water and glucose at 4.8 and 3.8 ppm, respectively, and resolved resonances from Glx and lactate at 2.4 and 1.4 ppm, respectively, in the human brain at 4 T. Here, at 7 T, we observed partially resolved resonances from fumarate and water at 6.5 and 4.8 ppm, respectively, and a resolved resonance from malate at 2.4 ppm. Since the fumarate and malate resonances show larger chemical shift differences from their immediately adjacent resonances than the water, glucose, Glx, and lactate resonances, and since the resolution of the ^2^H spectra scales linearly with magnetic field (34), then we would expect the fumarate and malate resonances to be resolved from each other and from the water resonance at 3 T, a field more widely used in the clinic. De Feyter et al. detected glucose and Glx at concentrations of up to 1.5 and 2 mM, respectively, in 20 × 20 × 20 mm^3^ voxels in the normal human brain at 4 T. Here, we detected fumarate and malate concentrations of around 5 mM. The expected decrease in sensitivity in going from 4 to 3 T is ∼40% (34), and therefore we expect to be able to detect fumarate and malate at 3 T, even if the fumarate dose has to be decreased for human studies.

In conclusion, this study has demonstrated that ^2^H spectroscopy and spectroscopic imaging of [2,3-^2^H_2_]fumarate metabolism can be used to detect tumor cell death posttreatment, before there are detectable changes in tumor size. Translation of this technique to the clinic could offer an approach to detecting early tumor responses to therapy and potentially to detect cell death in other conditions, such as acute kidney tubular necrosis and myocardial infarction.

## Supplementary Material

Supplementary File

## Data Availability

All study data are included in the article and/or *SI Appendix*.
