# Supplementary material for: Monitoring tumor cell death in murine tumor models using deuterium magnetic resonance spectroscopy and spectroscopic imaging
Source: Proc Natl Acad Sci U S A. 2021 Mar 16;118(12):e2014631118. doi: 10.1073/pnas.2014631118 (PMC8000230; doi:10.1073/pnas.2014631118)
Supplement: Supplementary File [file pnas.2014631118.sapp.pdf]

PNAS
www.pnas.org

Supplementary Information for
Monitoring tumor cell death in murine tumor models using deuterium magnetic resonance spectroscopy and spectroscopic imaging.

Friederike Hesse, Vencel Somai, Felix Kreis, Flaviu Bulat, Alan J. Wright, Kevin M. Brindle

K.M. Brindle
Email: kmb1001@cam.ac.uk

This PDF file includes:

Supplementary text
Figure S1
SI References

Supplementary Materials and Methods. Additional information for the materials and methods section.

Materials and Methods

Cell culture.

A murine lymphoma cell line (EL4) and two human cancer cell lines, a triple negative breast cancer cell line (MDA-MB-231) and a colorectal adenocarcinoma cell line (Colo205), were purchased from the American Type Culture Collection (ATCC) in March 2015 and were used within 5 to 10 passages from the original stocks. All cell lines tested negative for mycoplasma. Cells were grown in a humidified incubator at 37 °C and 5% CO2. Colo205 and MDA-MB-231 cells were transduced with a lentiviral vector that co-expressed the red fluorescent protein, mStrawberry, and firefly luciferase (19). Viability and cell number were assessed using a Vi-Cell counter (Vi-Cell XR, Beckman Coulter). Cells were harvested by centrifugation (EL4) or by centrifugation following trypsinization and washed with phosphate-buffered saline (PBS).

Tumor implantation
EL4, MDA-MB-231 or Colo205 cells (viability > 95%) were washed, resuspended in 0.2 ml chilled phosphate-buffered saline (PBS) or in a mixture containing 1:1 Matrigel (Corning) and PBS

(MDA-MB-231), and injected subcutaneously at 5 x 10^6, 7 x 10^6 and 10 x 10^6 cells respectively into the left flank of 8- to 12-week-old female C57BL/6J mice (EL4 tumors) or on the back and between the front legs of BALB/c nude mice (Colo205 and MDA-MB-231 tumors) (Charles River Laboratories). Baseline and post-treatment tumor volumes were measured daily using a caliper, with tumor volumes calculated according to the formula (length x $width^2$)/2.

Synthesis of disodium [2,3-$^2H_2$]fumarate and measurement of T_1

^1H and ^2H spectra acquired at 14.1 T using a high-resolution NMR spectrometer (Bruker Spectrospin Ltd.) confirmed product purity. The ^2H T_1 relaxation times of fumarate (1 mg dissolved in 600 μl water (10.4 mM)), HDO and also of DMSO-d6 (3 mM) and formate-d (5 mM), which were used as chemical shift and intensity standards, were measured at 37°C using an inversion recovery sequence (n = 16, TR = 5 s). The time between the 180 and the 90 pulses was varied between 0.01s and 5 s in 8 exponentially equidistant steps.

^2H MR spectroscopy measurements on media samples

EL4 cells in suspension were treated with 15 µM etoposide and Colo205 and MDA-MB-231 cells on plates with 10 pM MEDI3039 for 24 h. The cells were then washed in PBS, resuspended at 1 x 10^6 cells/ml in 10 ml culture medium, and 5 mM [2,3-$^2H_2$]fumarate was added. One ml samples were taken at the specified time points, centrifuged at 1000 g, and the supernatants transferred to 5 mm NMR tubes. DMSO-d6 (1.5 mM) was added to the samples as a reference and ^2H NMR spectra were acquired at 310 K using the ^2H coil of a 5-mm ^1H/broadband inverse detection probe in a 14.1 T high-resolution NMR spectrometer (Bruker Spectrospin Ltd.). Spectra were acquired using a 90° pulse, a repetition time (TR) of 2 seconds with a 2000 Hz spectral width into 1024 data points and were the sum of 1024 transients acquired over a period of 35 minutes. Spectra were phased, baseline corrected, and peak integrals calculated using Topspin (Bruker Spectrospin Ltd.). The amplitudes of the water, fumarate and malate resonances were normalized to the DMSO-d6 peak, after correction for the slight saturation of the DMSO-d6 resonance, in order to calculate concentrations (1, 2). Absolute concentrations were obtained by correcting for the numbers of deuterons per molecule. The rates of deuterated fumarate consumption and malate production and the rates of water labeling were determined by a linear least squares fitting of the first 2 hours of incubation using MATLAB (The Mathworks, Natick, USA).

MR spectroscopy and spectroscopic imaging in vivo

Animals were anesthetized by inhalation of 2 % isoflurane in air/O_2 (75 %/25 %, 2 L/min). Breathing rate and body temperature were monitored and body temperature maintained with a stream of warm air. Experiments were performed at 7 T (Agilent, Palo Alto, CA), where the ^2H resonance frequency is 46.007MHz. A 72 mm diameter birdcage volume coil was used for ^1H transmit and receive (Rapid Biomedical GMBH, Rimpar, Germany) and a home-built 10 mm diameter single-loop surface coil, located over the tumor, was used for ^2H transmit and receive. The tumors were localized in axial ^1H images acquired with a fast spin echo (FSE) pulse sequence (TR = 2000 ms, FOV = 39 x 39 mm with 256 x 256 matrix size, slice thickness = 3 mm, number of slices = 9). Serial ^2H spectra were acquired using a pulse-acquire sequence with a 2 ms BIR4 (3) adiabatic excitation pulse, with nominal flip angle of 67º, a TR of 140 ms and a spoiler gradient on the Z-axis to dephase any residual transverse magnetization. The carrier frequency of the excitation pulse was set to the resonance frequency of the natural abundance HDO peak and the pulse power adjusted to maximize the HDO signal. Spectra were zero- and first-order phase corrected, and the peaks fitted with the AMARES toolbox (4, 5). 3D chemical shift images (CSI) were acquired as described in (2) using the same excitation pulse followed by phase-encoding gradients encoding a 9 x 9 x 3 k-space matrix with a FOV of 27 x 27 x 27 mm^3. Data were acquired into 256 complex points with a bandwidth of 4 kHz. The k-space was sampled over 4328 transients with a repetition time of 140 ms. The center of k-space was sampled with 128 signal averages. All other k-space positions were sampled with fewer averages according to a Hamming window function, which reduces the side lobes of the point-spread function and improves the signal-to-noise ratio. The sampling order was randomized. A 4D

Fourier-transform yielded the 3D spectroscopic image, which was further processed similarly to the non-localized spectra.

^{2}H MR spectroscopy of erythrocyte suspensions
Erythrocytes were separated by centrifugation at 2000 g for 10 min from blood samples collected in *BD Microtainer®* blood collection tubes. The cells were then washed with oxygenated phosphate-buffered saline, containing 10 mM glucose, and then diluted to an haematocrit of ~40% in Krebs-Henseleit buffer (NaCl 118.5; $NaHCO_3$ 25.0; KCl 4.7; KH_2PO_4 1.2; $MgSO_4$ 1.2; glucose 11; $CaCl_2$ 2.4; mmol/L) which had been gassed with 95% O_2 / 5% CO_2 until the pH was approximately 7.4. To 0.6 mL samples in 5 mm diameter tubes were added 8 mM [2,3-$^2H_2$]fumarate and 5 mM formate-d6 and ^{2}H NMR spectra were acquired at 310 K using the ^{2}H coil of a 5-mm ^{1}H/broadband inverse detection probe. Spectra were acquired using a 90° pulse, a repetition time of 2 seconds with a 2000 Hz spectral width into 2048 data points and were the sum of 150 transients. Fourteen, 5 min spectra were acquired over a period of 65 minutes. Spectra were phased, baseline corrected, and peak integrals calculated using Topspin (Bruker Spectrospin Ltd.). Peak amplitudes were normalized to the deuterated formate peak in order to calculate concentrations.

Dynamic contrast-enhanced MRI
A separate cohort of EL4, MDA-MB-231 and Colo205 tumor-bearing animals (n = 3 per group) underwent dynamic contrast-enhanced (DCE)-MRI before and either 48 (EL4) or 24 (MDA-MB-231 and Colo205) hours after treatment with etoposide (EL4) or MEDI3039 (MDA-MB-231 and Colo205). Images were acquired at 9.4 T using a Bruker (Karlsruhe, Germany) console, an Agilent (Palo Alto, CA) magnet and a 40 mm diameter ^{1}H volume coil (Agilent, CA, USA). Baseline T_1 measurements were made using an inversion recovery-fast spin echo sequence (8 inversion times between 0.02 and 3 seconds, scan repeat time 10 seconds). The 90º and 180º pulses were determined using a series of spoiled-gradient echo variable-flip angle acquisitions. These were acquired at the same geometric positions as the DCE images (3 slices, 35 mm x 35 mm x 1 mm, 0.2 mm separation), which were also acquired using a spoiled gradient echo sequence (128 x 128 points, TR 20 ms, TE 2.5 ms, flip angle 12˚). A series of 500 images (2 averages, 5 seconds per set of 3 images) were acquired. Dotarem, at 200 μmoles/kg (Gadoteric acid, Guerbet), was injected via a tail vein after the 10th image. Signals from the image series were converted, on a pixel-by-pixel basis, to a contrast-agent concentration by assuming an R_1 relaxivity of the contrast agent of 2.7 $s^{-1}mM^{-1}$ (6).

Western blotting
Freeze-clamped tumor samples were homogenized in 10 μL/mg RIPA buffer (ThermoFisher Scientific) containing cOmplete mini EDTA-free protease inhibitor (Sigma Aldrich). Proteins were separated by SDS-PAGE and transferred to a nitrocellulose membrane using an iBlot 2 Dry Gel Transfer Device (ThermoFisher Scientific). Membranes were blocked with 1:1 Odyssey Blocking Buffer and Tris Buffered Saline (TBS) and incubated with a rabbit polyclonal IgG fumarase antibody (ThermoFisher) and a mouse monoclonal GAPDH antibody (Abcam) at 4°C overnight. Antibodies were detected using multiplexed IRDye secondary antibodies. The membrane was incubated with IRDye 800CW Goat anti-Rabbit IgG (LI-COR) and IRDye 680LT Goat anti-Mouse IgG antibodies (LI-COR) for 1 hour, with gentle shaking, at room temperature and analyzed using a LI-COR Clx scanner (LI-COR Biosciences).

Histology and immunohistochemistry
Sections of formalin-fixed paraffin-embedded tumors (10 μm) were stained with hematoxylin and eosin (H&E; ST020 Multistainer – Leica Microsystems) and for cleaved-caspase (CC3) using a rabbit monoclonal anti-CC3 antibody (Cell Signaling Technology) and a donkey anti-rabbit secondary biotinylated antibody (Jackson ImmunoResearch Laboratories) with a Polymer Refine Kit on an automated Bond platform (Leica Biosystems Ltd). Sections were also stained using TdT-mediated dUTP Nick- End Labeling (TUNEL) with a DeadEnd Colorimetric system kit (PromegaBenelux BV). Slides were scanned at 20x magnification with a resolution of 0.5 μm per

pixel on an Aperio AT2 (Leica Biosystems). Images were analyzed using a CytoNuclear v1.6 algorithm on HALO v3.0.311.293 (Indica Labs) to quantify percentage positivity for each stain.

Fig. S1.

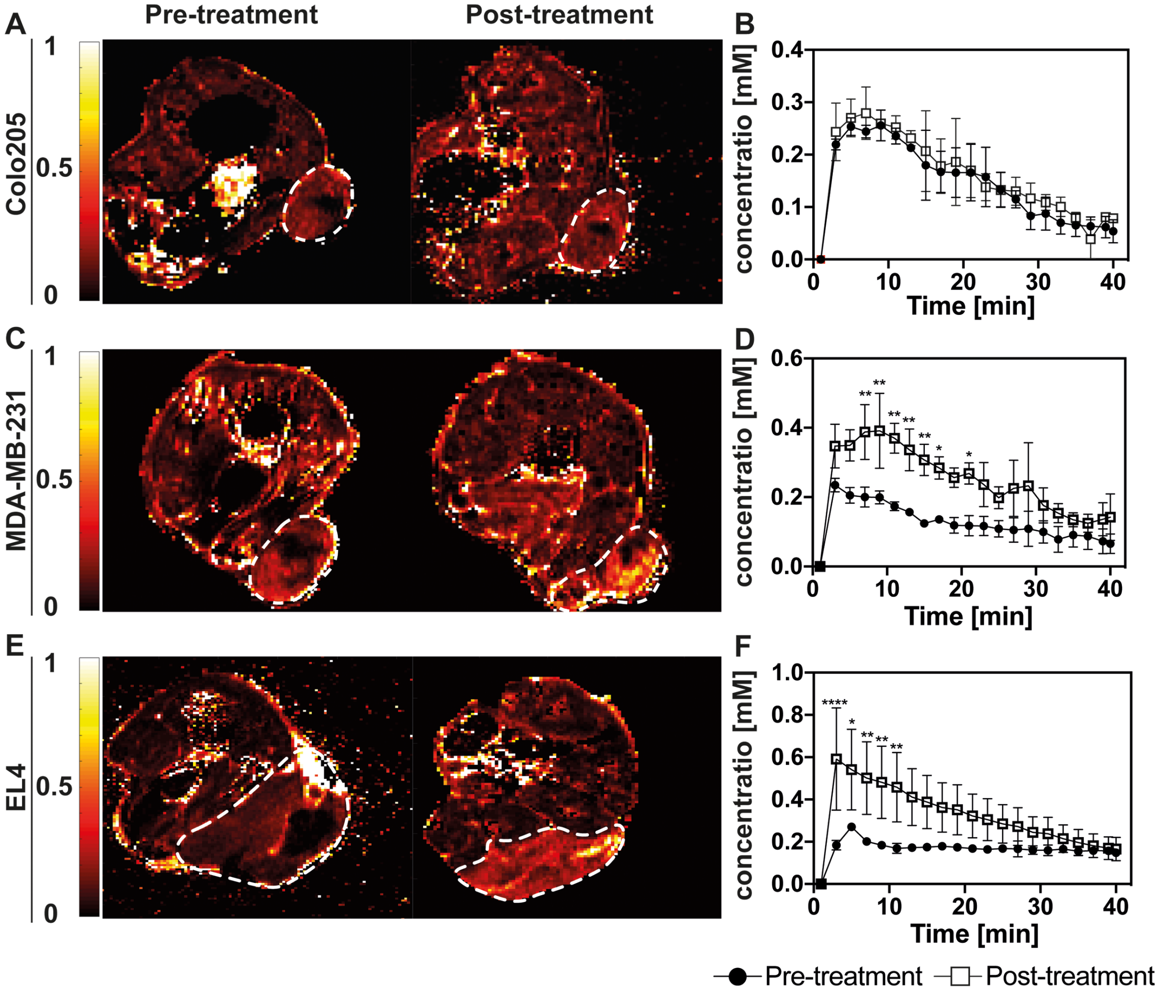

Dynamic contrast enhanced MRI measurements of Colo205 (n=3), MDA-MB-231 (n=3) and EL4 (n=3) tumor perfusion before and after treatment. (**A,C,E**) Representative color intensity maps of mean contrast agent concentration over the first three minutes following injection in Colo205 (**A**) MDA-MB-231 (**C**) and EL4 (**E**) tumors. The locations of the tumors are outlined by dotted white lines. (**B,D,F**) Estimated contrast agent concentration in tumor tissue at the indicated times after intravenous injection pre-and 24 h post MEDI3039 treatment (0.8 mg/kg) of Colo205 (**B**) and MDA-MB-231 tumors (**D**) and pre- and 48 h post etoposide treatment (67 mg/kg) of EL4 tumors (**F**), data are shown as mean ± SD, *P < 0.05, **P < 0.01, ****P < 0.0001).

SI References

1. De Feyter HM*, et al.* (2018) Deuterium metabolic imaging (DMI) for MRI-based 3D mapping of metabolism in vivo. *Sci Adv* 4(8):eaat7314.
2. Kreis F*, et al.* (2020) Measuring Tumor Glycolytic Flux in Vivo by Using Fast Deuterium MRI. *Radiology* 294(2):289-296.
3. Garwood M & DelaBarre L (2001) The return of the frequency sweep: designing adiabatic pulses for contemporary NMR. *J Magn Reson* 153(2):155-177.
4. Vanhamme L, van den Boogaart A, & Van Huffel S (1997) Improved method for accurate and efficient quantification of MRS data with use of prior knowledge. *J Magn Reson* 129(1):35-43.
5. Purvis LAB*, et al.* (2017) OXSA: An open-source magnetic resonance spectroscopy analysis toolbox in MATLAB. *PLoS One* 12(9):e0185356.
6. Lee H*, et al.* (2018) Quantitative Gd-DOTA uptake from cerebrospinal fluid into rat brain using 3D VFA-SPGR at 9.4T. *Magnetic resonance in medicine* 79(3):1568-1578.
